# Review of global neurosurgery education: Horizon of Neurosurgery in the Developing Countries

**DOI:** 10.1186/s41016-020-00194-1

**Published:** 2020-05-19

**Authors:** Y. Kato, B. S. Liew, A. A. Sufianov, L. Rasulic, K. I. Arnautovic, V. H. Dong, I. S. Florian, F. Olldashi, Y. Makhambetov, B. Isam, M. Thu, Ts. Enkhbayar, N. Kumarasinghe, A. H. Bajamal, S. Nair, S. Sharif, M. R. Sharma, J. A. Landeiro, C. G. Yampolsky, N. M. F. El-Ghandour, A. M. Hossain, S. Sim, S. Chemate, Hira Burhan, L. Feng, H. Andrade, Isabelle M. Germano

**Affiliations:** 1 Department of Neurosurgery, Fujita Health University Bantane Hospital, Nagoya, Japan; 2 Department of Neurosurgery, Hospital Sungai Buloh, Sungai Buloh, Selangor Malaysia; 3 Federal State-Financed Institution “Federal Centre of Neurosurgery” of Ministry of Health of the Russian Federation, Tyumen, Russia; 4 I.M. Sechenov First Moscow State Medical University, Moscow, Russia; 5 Faculty of Medicine, University of Belgrade, Belgrade, Serbia; 6 Semmes-Murphey Clinic and Department of Neurosurgery, University of Tennessee, Memphis, TN USA; 7 Neurosurgery Center of Viet Duc university hospital, Hanoi, Vietnam; 8 Department of Neurosurgery, University of Medicine and Pharmacy “Iuliu Hatieganu”, Cluj-Napoca, Cluj County Romania; 9 Department of Neurosurgery, University Hospital of Trauma, Tirana, Albania; 10 National Center of Neurosurgery, Astana, Kazakhstan; 11 Neurosurgical Centre, Yangon General Hospital, Yangoon, Myanmar; 12 Mongolian Neurosurgical Society, Ulaabaatar, Mongolia; 13 National Hospital of Sri Lanka, Colombo, Sri Lanka; 14 Department of Neurosurgery, Dr Soetomo General Hospital, Airlangga University, Surabaya, Indonesia; 15 Department of Neurosurgery, Sree Chitra Tirunal Institute for Medical Sciences and Technology, Thiruvananthapuram, India; 16 Institute of Postgraduate Studies and Medical Sciences, Liaquat National Hospital & Medical College, Karachi, Pakistan; 17 Department of Neurosurgery, TU Teaching Hospital, Kathmandu, Nepal; 18 Department of Neurosurgery, Universidade Federal Fluminense, Niterói, Brazil; 19 Department of Neurosurgery, Hospital Italiano de Buenos Aires, Buenos Aires, Argentina; 20 Department of Neurosurgery, Faculty of Medicine, Cairo University, 81 Nasr Road, Nasr City, Cairo, Egypt; 21 Bangladesh Society of Neurosurgeons, Dhaka, Bangladesh; 22 Khema Clinic, 18 Street, Phnom Penh, 528 Cambodia; 23 DNB Neurosurgery, Apollo Hospital, Chennai, India; 24 Institute of Neurosciences, Nobel Medical College and Teaching Hospital, Biratnagar, Nepal; 25 China International Neuroscience Institute, Beijing, China; 26 Department of Neurosurgery, University Medicine Mannheim, Mannheim, Germany; 27 Icahn School of Medicine, Mount Sinai Hospital, New York City, USA

**Keywords:** Global neurosurgical education, Developing countries

## Abstract

Globally, the discipline of neurosurgery has evolved remarkably fast. Despite being one of the latest medical specialties, which appeared only around hundred years ago, it has witnessed innovations in the aspects of diagnostics methods, macro and micro surgical techniques, and treatment modalities. Unfortunately, this development is not evenly distributed between developed and developing countries. The same is the case with neurosurgical education and training, which developed from only traditional apprentice programs in the past to more structured, competence-based programs with various teaching methods being utilized, in recent times. A similar gap can be observed between developed and developing counties when it comes to neurosurgical education. Fortunately, most of the scholars working in this field do understand the coherent relationship between neurosurgical education and neurosurgical practice. In context to this understanding, a symposium was organized during the World Federation of Neurological Surgeons (WFNS) Special World Congress Beijing 2019. This symposium was the brain child of Prof. Yoko Kato—one of the eminent leaders in neurosurgery and an inspiration for female neurosurgeons. Invited speakers from different continents presented the stages of development of neurosurgical education in their respective countries. This paper summarizes the outcome of these presentations, with particular emphasis on and the challenges faced by developing countries in terms of neurosurgical education and strategies to cope with these challenges.

## Background

Starting around a hundred years ago, the field of neurosurgery has experienced marked evolution on diagnostic modalities, microsurgical techniques, microsurgical adjuncts, and adjuvant therapies as part of the overall management of neurological diseases during the last few decades. Similar advances have also taken place in the sectors of neurosurgical training and education. To highlight these advances and their global scenario, a symposium was conducted at the World Federation of Neurological Surgeons (WFNS) Special World Congress Beijing 2019. Put forward by Prof. Yoko Kato, President of the Asian Congress of Neurological Surgeons (ACNS), the idea of this symposium was to discuss the current development of neurosurgical practice and education at a global scale. By inviting guest speakers from a number of different countries and representatives from various continental societies, this symposium provided an opportunity to overview the current facilities in the neurosurgical set ups, with a considerable emphasis on developing countries. This would enable formation of a central database that would later help formulate strategies to improve the neurosurgical development and resource facilities and to incorporate current technologies.

During this symposium, four keynote lectures were held on various aspects of education in neurosurgery, which included education for young neurosurgeons by Prof Yoko Kato; new strategies for young neurosurgeons’ education in Latin-America by Prof. Claudio G. Yampolsky; Horizon of neurosurgery in the developing countries by the WFNS President, Prof. Franco Servadei; and Neurosurgery Education in the World: Similarities, Differences, New frontiers by Prof. Isabelle M. Germano.

**Fig. 1:**
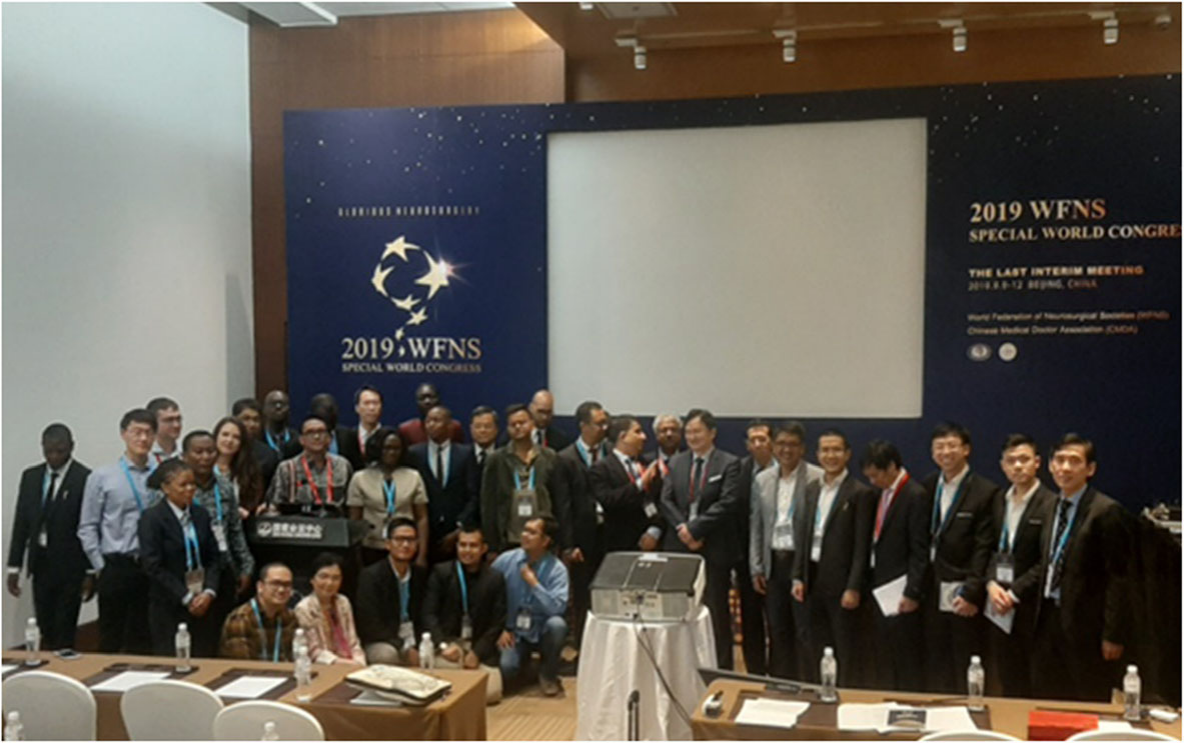
Group photo at the closing on day 1 of the symposium

Additional speakers are from Asian developing countries such as Tyumen, Vietnam, Mongolia, Indonesia, Malaysia, Myanmar, Philippines, Sri Lanka, China, India, Nepal, Pakistan, Thailand, Kazakhstan, Uzbekistan, Tajikistan, Saudi Arabia, and Cambodia. We also had invited speakers representing major neurosurgical societies, namely Latin American Federation of Neurosurgical Societies (FLANC), Continental Association of African Neurosurgical Societies (CAANS), American Association of Neurological Surgeons (AANS), Southeast Europe Neurosurgical Society (SeENS), the World Federation of Neurosurgery (WFNS) Education and Training Committee and speakers from different neurosurgical societies including Serbia, Bosnia-Herzegovina, Albania, Romania, Japan, Egypt, and Brazil and Venezuela. Names of all speakers and their respective countries are in Table 1. The group photograph is shown in Fig. 1.

**Table 1:** Invited speakers of the “Horizon of Neurosurgery in the Developing Countries” symposium 2019

Speakers	
Prof. Yoko Kato (Japan)	
Prof. Claudio G. Yampolsky (Italy)	
Prof. Franco Servadei (Italy)	
Prof. Isabelle M. Germano (USA)	
Prof. Hirotoshi Sano (Japan)	
Prof. Isam Elnour Baloul (Russia)	
Prof. Tsevegbat Enkhbayar (Mongolia)	
Prof. Abdul Hafid Bajamal (Indonesia)	
Prof. Hari Chandran (Malaysia)	
Prof. Myat Thu (Myanmar)	
Prof. Vanhe Dong (Vietnam)	
Prof. Eusebio L. Debuque (Philippines)	
Dr. Nilaksha Kumarasinghe (Sri Lanka)	
Prof. Bin Xu (China)	
Prof. Suresh Nair (India)	
Prof. Mohan R. Sharma (Nepal)	
Prof. Salman Sharif (Pakistan)	
Prof. Mahmood Qureshi (Kenya)	
Prof. Nakornchai Phuenpathom (Thailand)	
Prof. Kenan Arnautovic (USA)	
Prof. Hugo Andrade (Germany)	
Prof. Lukas Rasulic (Serbia)	
Prof. Yerbol Makhambetov (Kazakhstan)	
Prof. Fatos Olldashi (Albania)	
Prof. Ioan Stefan Florian (Romania)	
Prof. Gayrat Kariev (Uzbekistan)	
Prof. Ittichai Sakarunchai (Thailand)	
Prof. ATM Mosharef Hossain (Bangladesh)	
Prof. Amro F. Al-Habib (Saudi Arabia)	
Prof. Gap Legaspi (Philippines)	
Prof. Jose Alberto Landeiro	
Dr. Sachin Chemate (India)	
Prof. Sokchan Sim (Cambodia)	
Prof. Nasser El-Ghandou (Egypt)

## History of neurosurgery education from Asia and Africa

In *Mongolia*, the first four Mongolian neurosurgeons were trained between 1969 and 1970. However, there are many subspecialised neurosurgical procedures which are not yet available in Mongolia. These include functional neuro-endoscopy, transsphenoidal, and radiosurgery. There have been many training programs in conjunction with other countries since 1998 such as ACNS training program, WFNS educational courses, China International Neuroscience Institute (China-INI) young neurosurgeons’ training (Beijing), and the Foundation for International Education in Neurological Surgery (FIENS) Neurosurgical interactive training.

The first neurosurgical center in *Indonesia* was established in 1948, led by Prof. CH Lenshoek from the Netherlands. This was followed by Prof. Handoyo, Prof. Basoeki, Prof. Soewadji, Prof. Padmosantjojo, Prof. Iskarno, and Prof. Satyanegara who were the pioneers of Indonesian Neurosurgeons. The first national neurosurgical training was established in 1977 with twice yearly national board examinations. In the beginning of its development, the role of seniors from abroad was very important, including Prof. Tetsuo Kanno and Prof. Yoko Kato from Fujita Health University and Prof. Ramani from Mumbai either by giving fellowships or frequently making visits to Indonesia.

The first *Malaysian* neurosurgical unit was established in 1963 in Kuala Lumpur. The first Malaysian neurosurgeon who was trained in the USA was Dato’ Dr. Nadason Arumugam in 1969.

Local training program in *Myanmar* started in 2016, and currently, there are 16 neurosurgeons who graduated from the local training program with 36 neurosurgical trainees.

Early pioneers in neurosurgery in the *Philippines* were in 1930s. The Philippine Society of Neurological Surgeons founded in 1961 by eight fellows and followed by the establishment of Academy of Filipino Neurosurgeons in 1999. Philippines is the first country in the Southeast Asia to have a neurosurgery service. The first homegrown neurosurgeons were in 1980’s with small numbers involved in subspecialty services.

The *Sri Lankan* neurosurgical services were started back in 1956 with the establishment of 12 bedded neurosurgical wards by Dr. S.A. Cabral at the General Hospital, Colombo.

The first neurosurgical department in *India* was established in 1949 at the Christian Medical College, Vellore. Master training program in neurosurgery, Magister of Chirurgiae (MCh), or Master of Surgery—Neurosurgery (MCh—Neurosurgery) was begun in 1958.

The first neurosurgery was performed in *Nepal* in 1962 by Prof. Dinesh Nath Gongol. Nepalese Society of Neurosurgeons (NESON) was established in 2008 with 12 members.

Neurosurgery service in *Pakistan* was begun in 1951 with a spinal tumor resection surgery. Prof. Bashir Ahmed, Prof. Qazi, and Prof. OV Jooma were the pioneers who started neurosurgery and continued to train young surgeons to provide basic services. Functional and complex surgeries were performed in the 60’s. Pakistan Society of Neurosurgeons was established in 1987 with 22 neuro and general surgeons. This was registered with WFNS in 1987, and WFNS interim meeting was held in Lahore in 1989, under the chairmanship of Prof. Iftikhar Raja. Next few decades saw exponential growth of Neurosurgery and modernization. Local fellowship in Neurosurgery started, and training centers were established to cater for Pakistan and regional countries. Neurosurgery Fellowship exam is conducted by the Pakistani College of Surgeons, in various countries.

The Neurosurgical Association of *Thailand* was founded in 1982, followed by the establishment of the Royal College of Neurological Surgeons of Thailand in 2013.

The neurosurgical services in *Kazakhstan* have been in existence since 1964.

Prior to 1970, neurosurgical procedures in *Bangladesh* were performed by general surgeons. Prof. Rashid Uddin Ahmad is the founding father of neurosurgery in Bangladesh back in 1970. The first neurosurgery ward at that time was with 6 beds only. The 5-year neurosurgery master program started in 1997 in two institutions with additional of another institution in 2002.

In *Cambodia* prior to 2013, those pursuing neurosurgery need to do 3 years of general surgery, followed by 1 or 2 years fellowship overseas, mainly in France and other countries such as Vietnam, China, South Korea, and Japan. The local neurosurgery training started after that with a 4-year residency program.

Abu Al-Qasim Al-Zahrawi was the pioneer in neurosurgery in *Africa*, and neurosurgery began during the Egyptian and Babylonian era of medicine between the ninth and thirteenth centuries. The current priorities are to plan local training program, to obtain the necessary neurosurgery equipment.

## Current neurosurgical training program in Asia

Current data on availability of local training program and the year of its establishment; neurosurgery education system according to individual countries; and information regarding the population and capacity of their neurosurgery services was collected from the speakers representing their countries.

Following the symposium, information gathered from their presentation were summarized. A copy of tables with information related to the neurosurgery education and services were sent to all speakers within 2 weeks after the symposium for the purpose of verification of the data and to add on missing data. Literature reviews were performed for certain published data and facts.

The availability of the local neurosurgical training program and their year of establishment in the developing countries are tabulated (Table 2).

**Table 2:** The establishment of neurosurgery education in various countries

Country	Availability of local training program	Year of establishment
Vietnam	Yes	1995^a^
Mongolia	Yes	2014^b^
Indonesia	Yes	1977^c^
Malaysia	Yes	2001
Myanmar	Yes	2015^f^
Philippines	Yes	1975^e^
Sri Lanka	Yes	1979
India	Yes	1961^e^
Nepal	Yes	1999
Pakistan	Yes	1962^d^
Thailand	Yes	1985^d^
Uzbekistan	Yes	1997
Bangladesh	Yes	1997
Cambodia	Yes	2013
China	Yes	1953^d^

^a^Refer to [1]

^b^Refer to [2]

^c^Refer to [3]

^d^Refer to [4]

^e^Refer to [5]

^f^Refer to [6]

The neurosurgical education in each developing country involved in the study are tabulated in Table 3. Information includes the number of neurourgical training center, unformity in terms of the neurosurgical curriculum, prior general surgery training requirement, type of training (between structured program and apprenticeship), duration of training ( for both basic and sub-specialty training), the requirement for abroad fellowship program, recognized WFNS fellowship training centers, and the availability of local neurosurgical journals.

**Table 3:** Neurosurgery education system according to individual countries

Country		Vietnam	Mongolia	Indonesia	Malaysia	Myanmar	Philippines	Sri Lanka	India	Nepal	Pakistan	Thailand	Uzbekistan	Bangladesh	Cambodia
No. of training center		10^i^	1^b^	8	2	4^h^	10	3	59^g^	4^f^	22	11	4	4^k^	1
Uniform curriculum		No	No	Yes	No	*NK*	Yes^d^	Yes	Yes	Yes	No^a^	*NK*	*NK*	*NK*	Yes
Types of neurosurgery education	Apprenticeship		Yes							Yes^c^					
	Structured	Yes	Yes^b^	Yes	Yes	Yes	Yes^d^	Yes	Yes	Yes	Yes	Yes	Yes	Yes	Yes^j^
Gen surgery requirement (years)		No	Yes (3)^b^	Yes	No	No	Yes (1)		*O*	Yes					
Years of training		3	3	5.5	2 + 4	3^e1^	4–5	7	3^d1^ or 5^d2^	3^d1^	3–5^a^	5	3 or 4	5	4
Local sub specialization (years)		(3)	*NK*	Yes	No	(3)^e2^	*NK*	*NK*	Yes^g^	Yes	*NK*	*NK*	*NK*	*NK*	NK
Fellowship or oversea attachment (duration)		Yes	Yes	Yes (6 months)	No	Yes (3 months)^e^	Yes	Yes	Yes	Yes	Yes	Yes	Yes	Yes	Yes
WFNS accredited training center (number of center)		No	No	Yes (1)	Yes (1)	No	No	No	Yes (1)	No	Yes (1)	No	No	No	No
Own neurosurgery journal		No	No	Yes	Yes^l^	No	Yes^m^	Yes^m^	Yes	Yes^l^	Yes	No	No	Yes	No

*NK* not known, *O* optional

^a^Refer to [7]

^b^Refer to [2]

^c^Refer to [8]

^d^Refer to [4]

^d1^With general surgery

^d2^Without general surgery

^e^Refer to [9]

^e1^3-year M.Med.Sc in neurosurgery

^e2^3-year doctorate program (Dr.Med.Sc.)

^f^Refer to [10]

^g^Refer to [11]

^h^Refer to [6]

^i^Refer to [12]

^j^Online curriculum through University of Toronto

^k^Refer to [13]

^l^Journal of Neurosciences

^m^Journal of Neurology

The geographic and population data for the developing countries are tabulated in Table 4. The ratio of neurosurgeon with the geographic area and population are also presented.

**Table 4:** Neurosurgery services and their requirements in the developing countries

Country	Vietnam	Mongolia	Indonesia	Malaysia	Myanmar	Philippines	Sri Lanka	India	Nepal	Pakistan	Thailand	Uzbekistan	Bangladesh	Cambodia
Population (mil)	98	3.2	270	32,5	54	105	22	1,366	28	212	66.4	33	163	16
Area (km^2^)	331,212	1,600,000	1,919,000	330,803	663,327	300,000	65,610	3,287,000	147,181	881,913	513,120	447,000^a^	147,570	181,035
Neurosurgeon (2019)	600	27	376	174	23	145 (2018)	27	1693^b^	85	212	521	320	160	28
Neurosurgeons per 100,000 population ratio	0.612	0.844	0.139	0.535	0.043	0.138	0.123	0.124	0.304	0.100	0.785	0.970	0.098	0.175
Neurosurgeons per area (100 km^2^) ratio	0.182	0.002	0.020	0.053	0.003	0.048	0.041	0.052	0.058	0.024	0.102	0.072	0.108	0.015

^a^Refer to [14]

^b^Members of the neurosurgical society

In Sri Lanka, the basic neurosurgery education with three and a half years of general surgical training followed by 2-year local neurosurgery training and 2-years training abroad. In Uzbekistan, for those enrolled in Master’s program, the training period is 3 years while for those in the residency program, training period is 4 years. In India, the candidate for neurosurgery requires to have general surgery post master qualification. However, those who enter directly following basic medical degree require 6 years program. In Malaysia, training as medical officer for 2 years (minimal 1 year in neurosurgery) is required before enrolment in the 4 years Master’s program. Both Thailand and Bangladesh have 5-year program with Bangladesh adopting residency program.

In Indonesia, only government universities are allowed to conduct neurosurgical training program in Indonesia and must have at least one neurosurgical Professor, three PhD neurosurgeons with standard facility and minimal number of surgeries per year. The ratio of supervisor to student has been standardized to 1:3. The length of education is 11 semesters. There are currently 8 centers for Neurosurgery Education, and till date, Indonesia already has 376 neurosurgeons. In Malaysia, since the establishment of the first local neurosurgical training program by Prof. Dato’ Dr. Jafri Malin bin Abdullah in Universiti Sains Malaysia in 2001, a total of 78 neurosurgeons have been trained locally. The government of Myanmar has been training general surgeons in the country to perform neurotrauma surgery. So far, there are 160 general surgeons who have been trained. At the moment, there are only 4 well-equipped neurosurgical centers in Myanmar.

Due to limited number of neurosurgeons with high volume of workload, the training system in Vietnam has been structured to allow basic neurosurgical training for 3 years. After the training, those neurosurgeons can serve in district especially in neurotrauma. The unique regarding the service is that one neurosurgeon can bring with them an anaesthesiologist and nurses (operation theatre and ward) to local hospital for the duration of 3 months to run services and conduct teaching. Only after 2 years of service, those neurosurgeons will continue their training in sub-specialization training for another 3 years. The duration of training in general surgery rotation in India for a neurosurgery training program has been reduced to 3 months in view of global trend, while some neurosurgery programs, the general surgery rotation, have been removed completely from the syllabus. Sub-specialization is compulsory in India with 1 year training. Sub-specialization in Indonesia is for the duration of 2 years. In the Philippines, the current generation of millennium neurosurgeons (2000’s) is also known as the third generation of neurosurgeons with high numbers of subspecialty training. The Philippines Health Facility Development Plan 2017–2022 has identified the need to develop brain centers and acute stroke units in selected Department of Health hospitals.

The Kazakh National Center for neurosurgery which was opened in 2008 is one of the 50 neurosurgical specialized centers in Kazakhstan. This center has trained 41 residents in neurosurgery. The center’s future plans with the establishment of radiosurgery service, acquiring hybrid technologies, molecular studies, and organizing neurosurgical education program for central Asia. Re-credentialing is required for all neurosurgeons in Indonesia, Malaysia, and Uzbekistan. This process is conducted once every 5 years. Training abroad is available in many countries such as Tyumen, Vietnam, Myanmar, Philippines, Sri Lanka, Nepal, Pakistan, and Bangladesh. The duration of such training is about 20 months in Myanmar. In Sri Lanka, Fellowship of the Royal Colleges of Surgeons Specialty Examination in Neurosurgery (FRCS–SN) program started in 2018. In Bangladesh, such training is conducted with collaboration with the WFNS and ACNS.

In Mongolia, there have been many training program with other countries since 1988 such as ACNS training program, WFNS educational courses, China-INI young neurosurgeons’ training (Beijing), and FIENS Neurosurgical interactive training. At the moment, there has been lacking of local training program. The ratio of trainer to trainee is 1:3 in the neurosurgery master training program in Uzbekistan. Advance training is compulsory for all practicing neurosurgeons every 5 years. In Bangladesh, a 5-year residency program was started in Bangabandu Sheikh Mujib Medical University in 2010. The Bangladesh College of Physicians and Surgeons also offers Fellow of College of Physicians and Surgeons, FCPS (Neurosurgery) which is a neurosurgery fellowship program. The college has been training neurosurgeons from Bhutan and Nepal. They also have their own Bangladesh Journal of Neuroscience and Bangladesh Journal of Neurosurgery. In Nepal, the prerequisite to enrol into the 3-year neurosurgery residency (MCh) program is to have a masters in general surgery (Master of Surgery, MS, or equivalent) qualification.

## The population-based requirement of neurosurgeons

The unequal distribution of neurosurgeons globally is the main obstacle in ensuring accessibility to neurosurgical services. The developed countries are aiming to achieve a ratio of 1 neurosurgeon per 80,000 populations. However, the developing and under-developed countries are facing a bigger shortage of neurosurgeons with ratios of 1 neurosurgeon per 10 million population [15]. It is estimated that nearly 23,000 more neurosurgeons are required to be trained globally to treat at least five million essential neurosurgical cases each year [16]. Philippines, which is divided into 12 regions geographically, with just above 100 million population, is served by about 120 neurosurgeons, with ratio of near to one neurosurgeon to 0.8 to 1 million population. As of the year 2019, there are 14 neurosurgical centers with 27 neurosurgeons in Sri Lanka. The current deficit is 215 neurosurgeons as per recommended ratio of one neurosurgeon to 100,000 populations in Nepal. The number of graduates is about 20–25 per year. It is estimated to reach the target by 2030. Despite that, there is uneven distribution of neurosurgeons in different parts of the country. More than 40% neurosurgeons work in Kathmandu, serving only 14% of the total population.

Currently, there are 212 neurosurgeons serving 200 million populations with 415 trainees nationwide in Pakistan. The challenge for Pakistan Society of Neurosurgeons is to retain over 400 trainees by providing them incentives and post fellowship training. There is only 34.7% neurosurgeons working in 80 public hospitals in Thailand. In Uzbekistan, due to lack of instruments, lack of legislative for cadaveric courses, lack of simulation centers, and the trends of young neurosurgeons leaving country after graduation are problems faced by this country. Currently, in Cambodia, there are only 28 practicing neurosurgeons in the country of 16 million populations. Despite short number of neurosurgeons, 95% of spine surgeries are performed by neurosurgeons.

## Gaps in neurosurgery education in developing countries

The lack of training facilities such as cadaveric dissections, standardization of neurosurgery training program, and skills assessment were problems highlighted in a study on neurosurgery education in Pakistan published in 2016 [7]. This was addressed by the only WFNS recognized center from Karachi, which has held more than 40 hands-on workshops, conferences, and courses. Cadaver courses in anatomy, spine, endoscopy, skull-base, image guidance, and pituitary have helped young neurosurgeons and trainees in Pakistan and the region. More than a 100 faculty from around the globe has helped train these passionate surgeons.

Gaps in neurosurgery education are best to be tackled by geographical regions. Example is the CURE Hydrocephalus and Spina Bifida (CHSB) fellowship that offers an intensive pediatric subspecialty training program for trainees from the low and middle income countries (LMIC) especially from the African countries. The 8-week training period is held at the CURE Children’s Hospital of Uganda (CCHU) in Mbale, Uganda [17]. This is the best example to establish a neurosurgery education center within the geographic region to spur its development. This will help to reduce the high cost of living if the training has to be held in the high income countries (HIC). Due to more expensive, complicated and advanced neurosurgical equipment, and expensive drugs, it is becoming more difficult for developing countries to follow the neurosurgical standards in neurosurgical care achieved by developed countries [18]. The issue of lack of neurosurgical equipment needs to be addressed. Japan International Cooperation Agency (JICA) in their report on the project for construction of Mongolia–Japan teaching hospital in Mongolia in 2014 donated neurosurgical equipment to the hospital despite at the low priority [19].

In Uzbekistan, due to the lack of instrument, no legislative for cadaver lab, lack of simulation labs, and the brain drain of neurosurgeons have led to the increasing gap in neurosurgery education. There have been many training opportunities conducted by various organizations such as the WFNS and ACNS. The current trends of the general public demanding a senior neurosurgeon to treat their family members has resulted in decreasing training exposure for the young trainees and young neurosurgeons. Despite the high degree of delegation of some surgical procedures to the trainees in the University hospitals, the surgical outcomes in those institutions are better in comparison to the other institutions. There is a need to develop both hard skills and soft skills in the neurosurgery training program.

In Nepal, though imaging and lab facilities are widely available in urban areas, treatment of many neurosurgical conditions are heavily based on clinical judgement as frequent imaging is cost prohibitive for many. However, Nepal’s government support of 1000 USD for patients with head injury, spinal injury, and brain tumor is highly commendable. Also, accredited by the ACNS, the Institute of Neurosciences in the city of Biratnagar offers skull base and cerebrovascular training and fellowships to many young neurosurgeons and residents. It is a recently developed institute with state-of-the-art facilities including an intraoperative 3T magnetic resonance imaging (MRI), O-arm, and a navigable microscope that allows performing complex microsurgical procedures.

Global Initiatives in Sub Saharan Africa have been showing their impact on neurosurgical development in those countries. Two of seventeen sustainable development goals outlined by the World Health Organization (WHO) by 2030 are related to neurosurgery education. Goal no. 3 is to ensure healthy lives and promote well-being for all, at all ages. Goal no. 17 is aimed to revitalize a global partnership for sustainable development. Two out of ten non-communicable diseases, namely neoplasm and neurological disorders, are related to neurosurgeons in the global burden of disease categories. Meanwhile, transport injuries and other unintentional injuries are two out of four injuries in the global burden of diseases related to neurosurgical fraternity. In the list of 40 essential and emergency surgical procedures, ventriculoperitoneal shunt and craniotomy for traumatic brain injury are two commonly performed neurosurgical operations. The ratio of neurosurgeon to population is ranging from 1:2.7 million (Kenya) to 1:12 million (Ethiopia) with average of 1:7.9 million population for the African continent. Neurosurgical care in African continent has shown marked improvement since the establishment of local and international initiatives. These initiatives include those by the World Federation of Neurosurgery (WFNS) foundation, CURE Children’s Hospital in Uganda, Bethany kids Hospital in Kijabe, Kenya. Other initiatives include humanitarian missions (20 missions since 2001), outreach surgery, and training.

Training curriculum through regional and international collaboration includes those developed locally and through FIENS, which has been formally used as neurosurgery curriculum in Ethiopia and in South Africa. The concerted efforts have shown desirable result with the improvement in the ratio of neurosurgeon to population from 1:10.7 million in 2004 to 1:1.3 million in 2018. The Continental Association of Neurosurgical Societies (CAANS) plays important role in promoting African development initiatives. Other important partners in these global initiatives include the Asian Congress of Neurosurgery (ACNS), Yoko Kato’s Foundation (YKF), Foundation for International Education in Neurosurgery (FIENS), Country Government, Regional College of Surgeons of East, Central and Southern Africa (COSECSA), DUKE Global Neurosurgery and Neuroscience program, and Weill Cornell Tanzania Neurosurgery Project.

It is the responsibility of the senior neurosurgeons to teach the next generation of neurosurgeons especially from the developing countries, to decrease the disparity in world neurosurgical services level which including the effort to provide better equipment and resources to the developing countries. Currently there are high disparities in the distribution of 49,000 neurosurgeons worldwide. In 2019, over 170 courses have been organized by the WFNS. In 2017 to 2019, the WFNS has conducted more than 20 webinars, consensus conferences and more than 20 new initiatives. The organization has also published many guidelines, book, white papers and newsletters. Currently there are many publications related to traumatic brain injury by countries with low burden of such diseases in comparison with countries with high burden of the disease with low volume of such publications. The effort to improve access to journals for publication among neurosurgeons from the low and middle income countries as to counter the disparity has been implemented. There is a need for resourceful countries to help less resourceful countries in term of organizing hands-on courses, on-site training, and financial support.

The importance of training abroad for young neurosurgeons among the developing countries has been the priority for the SeENS. The SeENS Silk road courses have trained about 100 young neurosurgeons from different many developing countries around the world. There has been increasing number of neurosurgery female residents from 8% in 1989 to 16% in 2016 in the USA. Despite that, there is low percentage of board-certified women neurosurgeons with only 6.1% in 2016. The neurosurgery education has been achieved with many programs such as boot camps, junior and senior resident courses, and neurosurgery fellowships.

The neurosurgery education should comprise technique of stabilizing their hands during surgery and to review the recordings of their previous surgeries to identify all unnecessary steps. Those doing the difficult things in a difficult way are an average surgeon, while those doing the easy things in a difficult way is a poor surgeon. Those who are doing the difficult things in an easy way are to be considered the master. A sound knowledge in anatomical landmarks may substitute the use of neuro-navigation system. The current generation of neurosurgeons should reduce their dependency on technology and always use anatomy landmarks. A proper risk explained to patient when required intraoperative neurophysiological monitoring is not available.

There are three types of hospital providing emergency services with 5 tertiary hospitals in Albania. There were only two department of neurosurgery in Albania with neurosurgery service limited in the capital city, with no neurosurgery in other regional hospitals. The limited neurosurgery service has been complimented with telemedicine in this country. Its use has resulted into more than two third of patients remained at the regional hospital, and the remaining was transferred to the tertiary hospital with neurosurgery service.

In a study done in 2016 among neurosurgical training centers in Pakistan, few indicators regarding the neurosurgery education was highlighted. The mean duration of training reduced from 3.8 years in 2010 to 2.8 years in 2016. Despite improvement in terms of internet facility (from 60 to 87%), there were declines in terms of basic science teaching session, mortality and morbidity meetings, and neuro-radiology and neuropathology meetings between the two studies. The main references for neurosurgery education shifted from Youman’s textbook of neurosurgery in 2010 to Greenberg’s handbook of neurosurgery [7].

A standard and minimal requirement in global neurosurgery education syllabus is required in order to arrest the declination in the quality of neurosurgery education and training. Objective tracking of resident performance parameters by semester, complimented with feedback from the educators and 360 degree assessment are equally important than the duration of training [20]. Most of the models of training initiated by HIC for the LMIC are not self-sustainable financially. Those programs are funded by a variety of donors, foundations, grants, and partnerships [2, 17]. A self-reliance program must be built in those LMIC with collaboration between the WHO and the governments.

## Future aspirations in neurosurgery education

The training program for neurosurgery is expensive and requires highly committed teachers and high volume and varieties of cases. The initiatives carried out by certain high income countries (HIC) to train neurosurgeons from low and middle income countries (LMIC) since the past 10 years has been very successful in accelerating the neurosurgery education and training. More important is the long-term mentorship that has been established between mentors from HIC with mentee from the LMIC [21]. Besides having been trained as a competent neurosurgeon, one should also be trained as a good neurosurgery teacher. According to Prof. Edward C. Benzel in his paper, we retain 90% of what we do or teach while we only retain 50% of what we hear and see [20].

The Ministry of Health of Russian Federation has introduced the state-of-art facilities of neurosurgical services and neurosurgical training in the city of Tyumen called the Federal Centre of Neurosurgery (FCN). The center is headed by the Chief Physician and Medical Director Albert Sufianov, professor of neurosurgery and the chairman of the academic department of neurosurgery at Sechenov University (Moscow). Regarding the neurosurgical education, the Center had incorporated training as one of its major goals, since its establishment in 2010. To achieve that goal, an initiative lead by Prof. Albert Sufianov and Prof. Yoko Kato was created in 2013, the main objective of which was to establish educational courses of Asian Congress of Neurological Surgeons (ACNS) and to prepare the FCN to be a destination for neurosurgical education and training of young specialists coming from developing countries. This initiative was implemented on October 3–4, 2013, when the FCN became the venue for the First ACNS Educational Course. Since then, seventy-four training activities have been conducted in the Center under Prof. Sufianov’s supervision, such as conferences, hands-on workshops, and seminars covering most of the neurosurgical topics, such as skull base surgery, pediatric neurosurgery, spinal neurosurgery, and neuro-oncology. The profile of the lecturers teaching at these events represents the whole globe, with remarkable contribution of Japan, Brazil, Germany, the USA, the UK, and 33 more countries. Among the total number of 1532 trainees, who attended these programs, there were 214 (14%) from developing countries, including post-soviet states and far-abroad countries (in total 27 countries). Apart from short educational events, the Department of Neurosurgery of Sechenov University offers residency and individual fellowship programs at the venue of the Federal Centre of Neurosurgery, which is a clinical site for the department. Apart from the Russian citizens, the residents from Kazakhstan, Kyrgyzstan, Tajikistan, Turkmenistan, Saudi Arabia, Sudan, and Columbia have studied or are still taking their courses here. At present, the FCN has created excellent facilities for comprehensive education, both short-term and long-term, for residents and practicing neurosurgeons. The unique feature of the Center is the integrated 3D anatomic simulation and vet laboratory allowing training in close-to-real operative conditions for both microsurgical and endoscopic approaches. Exercises in the laboratory are complemented with the possibility to observe 3D live surgeries in a perfectly equipped conference hall. These two major aspects ensure fast and effective training results. Taking into account combination of teaching methods and facilities, the FCN can make a significant contribution to improving the level of international neurosurgical education, especially regarding developing countries, and might serve as a model of facility successfully combining clinical practice, research, and education.

Prof. Yoko Kato in 2017 stated regarding the training of neurosurgeons which are very different from other profession. The training should not be limited with acquiring textbook knowledge and laboratory work, but also skillful in the surgical and non-surgical management of patients with neurological diseases.

The Latin American Federation of Neurosurgical Societies (FLANC) is a Continental Federation. Twenty-three societies, 2 regional and 3 extracontinental societies, are part of it. The official numbers may not accurately show the actual number of neurosurgeons as many of them did not join any of the country societies. There are over 10,000 neurosurgeons in Latin-America but many of them have not got there country society membership. Competency-based medical education has been practiced among FLANC members. The neurosurgical training is divided into 5 levels. The lowest level during the first and second year of residency is to acquire fundamental knowledge of both neurophysiology and neuroanatomy (“Know” phase). Mid-level residents are required to apply the knowledge into practice. The surgical skills and ability to make accurate diagnosis in neurosurgical disease are essential in this level (“Knows how” phase). As a senior resident in the third phase, one is required to perform surgical procedures under supervision (“Shows How” phase). The fourth phase is for unsupervised practice (“Does” phase). The final level of training is becoming innovator in the neurosurgical field (“Does better” phase). FLANC has been using new educational tools as social media and internet. More than 200,000 visitors entered our web page, offered neurosurgeons more than 80 webinars with more than 6000 accesses. The workplace has been developed through a Facebook association and we have more than 1500 neurosurgeons registered and interacting. Grants and scholarships have been offered for residents and young neurosurgeons for short visits in FLANC reference centers. The first FLANC-European Association of Neurosurgical Societies (EANS) Training Course was held in Rosario, Argentina, on Neuro-oncology and skull base. Eight European and 18 Latin-American professors joined our course. Eighty-eight residents and young neurosurgeons attended it. FLANC is planning to continue with other courses in different cities in Latin-America in order to contribute to education among our neurosurgeons.

The Southeast Europe Neurosurgical Society (SeENS) which was formed in 2012 held its first congress of SeENS in 2013, with subsequently another 3 congresses have been successfully organized. In 5-year period (2012-2017), SeENS has become an affiliated member of the European Association of Neurosurgical Societies (EANS) and full member of the World Federation of Neurosurgical Societies (WFNS). Currently SeENS is gathering neurosurgeons from 14 countries of the Southeast Europe region and Turkey. The SeENS’ educational programs include live surgeries, WFNS courses, workshops, masterclasses, and scientific meetings. The SeENS International Neurosurgery course with the term I course II was held recently this year in Zegreb, Croatia.

The future vision for the Bangladesh Society of Neurosurgeons is to establish dedicated subspecialty facilities like pediatric neurosurgery, neurovascular surgery, neuro-oncology, skull base neurosurgery, neurotrauma, functional, spine surgery, and endoscopic neurosurgery. Egyptian Society of Neurological Surgeons (ESNS) aspires to have greater involvement of African countries in local training and greater cooperation in terms of technical support and funding.

Simulator learning, e-learning, webinar, hands-on workshop, boot camp, and symposium have been existing as part of neurosurgery education tools. Simulators that mimic real-life operative experience and challenges especially in endovascular are important component of training [20]. Current development in simulator expands to other neurosurgery subspecialty including neuro-oncology with 3D visualization and tactile feedback. Digital education is an emerging tool in neurosurgery education with free and low-cost mobile content. These digital contents with high educational impact such as WFNS Young Neurosurgeons Forum Stream, Brainbook, NeuroMind, UpSurgeOn, The Neurosurgical Atlas, Touch Surgery, The 100 UCLA Subjects in Neurosurgery, Neurosurgery Survival Guide, and Neurosurgical.TV have become important educational materials [22].

Another milestone in the contribution of neurosurgical training and education is the formation of the Asian Medical Students and Residents Society for Neurosurgery (AMSRS) which was founded back in March 2018 as the student’s wing of the ACNS under the leadership of Dr. Hira Burhan. This society aims to bridge the gap between neurosurgical consultants and trainees, residents with a particular interest for medical students who are willing to pursue neurosurgery as a career. This society has by far conducted more than 200 online symposiums, available for free viewership around the world. Moreover, the society has been playing a major role in collaborating with WFNS, EANS, ACNS, and other societies by offering scholarships and recommendations for YNS and medical students. It has also organized its first workshop and conference at the ACNS center in Biratnagar, where, for the first time, a dedicated session for medical students was conducted to provide them a platform for neurosurgical exposure. In the coming years, the AMSRS intends to expand its activities for medical students and residents by opening opportunities for clinical rotations, fellowships, and publications in prestigious journals.

Boot camps involving trainees from both developed and developing countries help in establishing a standardized training in basic neurosurgical competencies by providing fundamental didactic and technical exposure [6].

Global neurosurgery is another initiative started few years ago in order to encourage trained neurosurgeons to perform services in other countries and places requiring neurosurgical services but with no rewards in term of academic promotions, no special funding, and no reimbursement for the work done [21, 23]. Those who have direct and indirect involvement in neurosurgery education should prioritize excellence in education [20]. The establishment of the Neurosurgery Research and Education Foundation (NREF) has been funding more than 200 grants for promising work by young investigators [24]. Besides that, the American Association of Neurological Surgeons (AANS) has also established Medical Student membership to the AANS. This helps to introduce and promote their involvement in neurosurgical research among medical students, and eventually to facilitate their later careers in neurosurgery [25].

To achieve true global neurosurgery, the concept of an international consensus in ensuring sound knowledge and practical competency through a world certifying body is essential. The contributions from all neurosurgical societies across the world to model their training curriculum based on an examination model with high standards and international validity should be the next strategy by the world organization in neurosurgery [26]. The needs for sub-specialization have been catalyzed by the exponential growth of neurosurgical knowledge [10]. Sub-specialization in neurosurgery itself helps in the innovation and development of neurosurgery.

## Conclusion

Most developing countries still lack of adequate number of medical workforces in the care of neurosurgical patients, neurosurgical facilities, and neurosurgical training. The shortage of neurosurgical facilities includes dedicated neurosurgical beds, intensive care unit beds, and hospital with adequate neurosurgery capacity. Other facilities include computed tomography, magnetic resonance imaging, and specialized neurosurgical equipment. There are also identical problem with neurosurgeons concentrated in the urban compared to suburban with high discrepancy. The poor co-operation between neurosurgical societies, low allocation, and poor development in neurosurgery education program by the governing bodies in the developing countries have been identified as the main factors for the slow improvement in the neurosurgery services within those countries. The current initiatives carried out by various institutions, organizations, societies, and individuals especially those from HIC have been resulting in the increase number of trained neurosurgeons in the LMIC. Self-reliance with dedicated neurosurgery education and training should be established in all countries in order to ensure sustainability in neurosurgery services. The equality in the access to the neurosurgical treatments by all individuals should be the ultimate aim in structuring neurosurgery education, human resources, and facilities in all countries across the globe.

## Data Availability

Data sharing is not applicable to this article as no datasets were generated or analyzed during the current study.

## Abbreviations

WFNS: World Federation of Neurological Surgeons
ACNS: Asian Congress of Neurological Surgeons
FLANC: Latin American Federation of Neurosurgical Societies
CAANS: Continental Association of African Neurosurgical Societies
AANS: American Association of Neurological Surgeons
SeENS: Southeast Europe Neurosurgical Society
China-INI: China International Neuroscience Institute
FIENS: Foundation for International Education in Neurological Surgery
MCh: Magister of Chirurgiae
FRCS–SN: Fellowship of the Royal Colleges of Surgeons Specialty Examination in Neurosurgery
FCPS: Fellow of College of Physicians and Surgeons
MS: Master of Surgery
CHSB: CURE Hydrocephalus and Spina Bifida
LMIC: Low and middle income countries
CCHU: Children’s Hospital of Uganda
HIC: High income countries
JICA: Japan International Cooperation Agency
MRI: Magnetic resonance imaging
WHO: World Health Organization
CAANS: Continental Association of Neurosurgical Societies
YKF: Yoko Kato’s Foundation
COSECSA: Country Government, Regional College of Surgeons of East, Central and Southern Africa
FCN: Federal Centre of Neurosurgery
EANS: European Association of Neurosurgical Societies
NREF: Neurosurgery Research and Education Foundation
AANS: American Association of Neurological Surgeons
AMSRS: Asian Medical Students and Residents Society for Neurosurgery
