# Correction to: Review of global neurosurgery education: Horizon of Neurosurgery in the Developing Countries

**DOI:** 10.1186/s41016-020-00209-x

**Published:** 2020-08-24

**Authors:** Y. Kato, B. S. Liew, A. A. Sufianov, L. Rasulic, K. I. Arnautovic, V. H. Dong, I. S. Florian, F. Olldashi, Y. Makhambetov, B. Isam, M. Thu, Ts. Enkhbayar, N. Kumarasinghe, A. H. Bajamal, S. Nair, S. Sharif, M. R. Sharma, J. A. Landeiro, C. G. Yampolsky, N. M. F. El-Ghandour, A. M. Hossain, S. Sim, S. Chemate, Hira Burhan, L. Feng, H. Andrade, Isabelle M. Germano

**Affiliations:** 1 Department of Neurosurgery, Fujita Health University Bantane Hospital, Nagoya, Japan; 2 Department of Neurosurgery, Hospital Sungai Buloh, Sungai Buloh, Selangor Malaysia; 3 Federal State-Financed Institution “Federal Centre of Neurosurgery” of Ministry of Health of the Russian Federation, Tyumen, Russia; 4 I.M. Sechenov First Moscow State Medical University, Moscow, Russia; 5 Faculty of Medicine, University of Belgrade, Belgrade, Serbia; 6 Semmes-Murphey Clinic and Department of Neurosurgery, University of Tennessee, Memphis, TN USA; 7 Neurosurgery Center of Viet Duc University Hospital, Hanoi, Vietnam; 8 Department of Neurosurgery, University of Medicine and Pharmacy “Iuliu Hatieganu”, Cluj-Napoca, Cluj County Romania; 9 Department of Neurosurgery, University Hospital of Trauma, Tirana, Albania; 10 National Center of Neurosurgery, Astana, Kazakhstan; 11 Neurosurgical Centre, Yangon General Hospital, Yangoon, Myanmar; 12 Mongolian Neurosurgical Society, Ulaabaatar, Mongolia; 13 National Hospital of Sri Lanka, Colombo, Sri Lanka; 14 Department of Neurosurgery, Dr. Soetomo General Hospital, Airlangga University, Surabaya, Indonesia; 15 Department of Neurosurgery, Sree Chitra Tirunal Institute for Medical Sciences and Technology, Thiruvananthapuram, India; 16 Institute of Postgraduate Studies and Medical Sciences, Liaquat National Hospital & Medical College, Karachi, Pakistan; 17 Department of Neurosurgery, TU Teaching Hospital, Kathmandu, Nepal; 18 Department of Neurosurgery, Universidade Federal Fluminense, Niterói, Brazil; 19 Department of Neurosurgery, Hospital Italiano de Buenos Aires, Buenos Aires, Argentina; 20 Department of Neurosurgery, Faculty of Medicine, Cairo University, 81 Nasr Road, Nasr City, Cairo Egypt; 21 Bangladesh Society of Neurosurgeons, Dhaka, Bangladesh; 22 Khema Clinic, 18 Street, Phnom Penh, 528 Cambodia; 23 DNB Neurosurgery, Apollo Hospital, Chennai, India; 24 Institute of Neurosciences, Nobel Medical College and Teaching Hospital, Biratnagar, Nepal; 25 China International Neuroscience Institute, Beijing, China; 26 Henan Provincial People’s Hospital, Juha Hernesniemi International Center for Neurosurgery, University of Zhengzhou, Zhengzhou, China; 27 Icahn School of Medicine, Mount Sinai Hospital, New York City, USA

**Correction to: Chin Neurosurg J 6, 19 (2020)**

**https://doi.org/10.1186/s41016-020-00194-1**

Following publication of the original article [1], the author H. Andrade identified an error of his affiliation.

The correct affiliation is: Henan Provincial People’s Hospital, Juha Hernesniemi International Center for Neurosurgery, University of Zhengzhou, Zhengzhou, China
